# Determination of human papillomavirus type in archival tissue specimens of invasive cervical cancer using molecular mapping and *E6/E7*-based polymerase chain reaction

**DOI:** 10.1371/journal.pone.0265996

**Published:** 2022-04-05

**Authors:** Jinichi Sakamoto, Mayumi Saito, Shitai Zhang, Masahiro Takakura, Hiroaki Takagi, Toshiyuki Sasagawa

**Affiliations:** Department of Obstetrics and Gynecology, Kanazawa Medical University, Uchinada, Ishikawa, Japan; Azienda Ospedaliero Universitaria Ospedali Riuniti di Ancona Umberto I G M Lancisi G Salesi, ITALY

## Abstract

In our previous study, an L1-based human papillomavirus (HPV) test using liquid-based cytology revealed that some invasive cervical cancers (ICC) exhibited multiple HPV types or harbored no HPV DNA. Here, molecular mapping of formalin-fixed paraffin-embedded cancer tissue specimens from the same patients were conducted to confirm these observations. Among 377 ICC cases, 73 eligible specimens (9 positive for multiple HPV types, 16 negative for HPV, and 48 positive for a single HPV type from the previous study) were reexamined by manual microdissection of cancer lesions, then subjected to HPV genotyping using the uniplex *E6/E7* polymerase-chain-reaction method to detect all high-risk and potentially high-risk HPV types. The HPV typing results were confirmed in 52 of 73 cancer cases; among the 21 remaining cases, 15 were discordant and 6 were partially concordant. In total, 8 of 16 (50%) HPV-negative samples became positive; 6 were positive for HPV16 and 2 were positive for HPV67. Moreover, two samples previously positive for HPV6 and HPV53 were negative for HPV. All nine cancers with multiple HPV types were found to harbor only a single HPV type. In total, 63 cancer tissues exhibited a single HPV type. HPV16 and HPV18 were detected in squamous cell carcinoma (SCC) and adenocarcinoma (ADC). Alpha-5 (HPV82), -6 (HPV56), and -9 (HPV31/52/67) HPV types were detected in SCC, whereas Alpha-7 (HPV59/68) types were detected in ADC and adenosquamous carcinoma (ADSCC). These findings suggested that the different HPV types induced different histological cancers. Furthermore, all SCCs and 10 of 11 usual-type ADCs were positive for high-risk HPV types, supporting the use of HPV screening for the detection of these cancers and associated premalignant lesions. HPV16 is likely to remain undetected in some cervical cancer tissues because of low viral-copy-numbers. Putative high-risk HPV types (e.g., HPV67 and HPV82) might be high risk in Japan.

## Introduction

Cervical cancer is the fourth most common malignancy in women, with 570,000 new cases reported in 2018 (constituting 6.9% of all cancer cases in women worldwide) [1]. In total, 13,069 new cervical cancer cases and 4,175 deaths were reported in 2015 in the United States, while 10,776 new cases and 2,812 deaths were recorded in Japan in the same year [2, 3]. We estimated that there were 3,500 deaths based on analyses of publicly available case reports of histologically undetermined cervical cancer in 2019. The high incidence of cervical cancer among young women in Japan represents a major social problem that must be addressed.

Although the Pap test contributed to decreases in the incidence and mortality of cervical cancer up to 1990, its low sensitivity for premalignant and malignant lesions in young women is widely recognized [4]. This prompted the incorporation of human papillomavirus (HPV) tests into cervical cancer-screening programs in Europe, as well as their clinical approval in the United States. Several randomized controlled trials revealed that Pap and HPV co-testing is not optimal because it is associated with higher costs and more unnecessary examinations [5]. Therefore, the primary HPV test is now considered the most cost-effective screening method for cervical cancer.

However, there remain limitations associated with the use of commercially available HPV tests for primary screening. Previous studies reported that the HPV genotype matching rate is generally low, with an approximate range of 77% to 84% among different HPV tests [6, 7]. Arroyo *et al*. [8] reported that, upon reexamination of 92 HPV-negative invasive carcinoma cases, high-risk HPV types were detected in 48 (52.2%). Different sensitivities or analysis methods among HPV tests can contribute to conflicting results. Recently approved HPV tests recognize HPV66 as a high-risk type; however, it is not detected by standard hybrid capture tests. Additionally, HPV52 is nearly undetectable in high-grade intraepithelial lesions by the Roche Linear Array HPV genotyping test, or by HPV tests using GP5+/6+ primers [9, 10].

In 2009, the International Agency for Research on Cancer suggested that 12 HPV types (HPV16/18/31/33/35/39/45/51/52/56/58/59) should be classified as high-risk types (Group 1), while HPV68 should be classified as a probable high-risk type (Group 2A). Eleven other HPV types (HPV26/30/34/53/66/67/69/70/73/82/85) were classified as possible high-risk types (Group 2B). Geographic differences in the prevalence of certain HPV types have been reported in various studies. The incidence of cervical cancer caused by HPV16 or HPV18 has been reported at 82% in Europe and the United States, compared with 68% in East Asia [11, 12]. Several studies reported that the prevalence of HPV16 and HPV18 were approximately 60% in Japan, while the prevalence of HPV52 and HPV58 are higher in East Asia than in Western countries [12–14]. Differences in the most prevalent HPV types among areas might not result from geographic differences alone. Various epidemiological studies conducted in Europe and the United States identified HPV types using GP5+/GP6+ polymerase chain reaction (PCR), MY09/11-PCR, and INNO-LiPA, whereas Southern blot hybridization, L1C/PCR [15], and LCR*E7-E6* PCR [16] were used in Japanese studies. L1C/PCR employs a pair of consensus primers that target HPV *L1* DNA, whereas LCR*E7-E6* PCR includes four consensus primers that target *E6* and *E7* DNA. Moreover, many commercial HPV tests target *L1*, which might be “lost” in some cancer tissues because of integration into the host genome [17, 18]. Among newer HPV tests (e.g., Aptima) that detect HPV *E6* and *E7* mRNA, some exhibit higher specificity for the detection of high-grade squamous intraepithelial lesions compared to more established HPV tests, including the hybrid capture and Cobas HPV tests [7, 19–21]. Therefore, geographical differences in HPV prevalence, as well as technical differences among tests, should be considered to ensure that the most appropriate HPV test is selected for primary screening.

We previously examined the prevalence of HPV types in 371 invasive cervical cancer (ICC) cases using a new HPV test (Genosearch-31 with additional 4 types) for the detection of 13 high-risk HPV types and 8 possible high-risk types (Group 2B; HPV26/30/53/66/67/70/73/82) [22]. This assay can detect the *L1* gene of 31 HPV types with equal sensitivity because each HPV is amplified by PCR using type-specific primers. Using this test, multiple HPV types were identified in cancer tissues from 31 (8.4%) cases. When cases with multiple HPV infections were excluded, the combined prevalence of HPV16 and HPV18 was 65.4%, which was higher than the values reported in all previous Japanese studies (50.1–57.5%) [13, 14]. The high rates of HPV16 and HPV18 in our previous study are consistent with the increases in adenocarcinoma (ADC) and adenosquamous cell carcinoma (ADSCC) cases, considering that these cancer types are mainly induced by HPV16 (Alpha-9) or HPV18 (Alpha-7) [22, 23]. Our previous study also demonstrated that the combined prevalence of the high-risk types HPV39 (Alpha-7) and HPV51 (Alpha-5) was 0.7%, while there were no cases of HPV35. However, a possible high-risk type, HPV67 (Alpha-9), was detected in 1% of cervical cancer cases, while HPV53 (Alpha-6) was detected in 0.7%, and HPV69 (Alpha-5) and HPV70 (Alpha-7) in 0.3%. Another possible high-risk type (HPV 82; Alpha-5) was detected in four cases of cervical intraepithelial neoplasia grade 3 (CIN3). These results suggest that HPV types belonging to Alphas-5, -6, -7, and -9 are generally oncogenic; thus, the classifications of high-risk HPV types for the Japanese population should be reconsidered. However, possible high-risk type HPV66, which can be detected with new commercial HPV tests, was not identified in Japanese cervical cancer patients.

In this study, we investigated HPV genotypes using a new HPV test, the uniplex *E6/E7* PCR, which can detect *E6* or *E7* DNA of 39 HPV types, including 13 high-risk HPV types and all possible high-risk HPV types (HPV26/30/34/53/66/67/69/70/73/82/85) [24]. Furthermore, the assay can detect all high-risk and possible high-risk HPV types with equal sensitivity. To our knowledge, this is the first study to extensively examine HPV genotypes through the combined approach of tissue microdissection of formalin-fixed paraffin-embedded (FFPE) specimens and the new *E6/E7*-based PCR method.

## Materials and methods

### Materials

**Fig 1:**
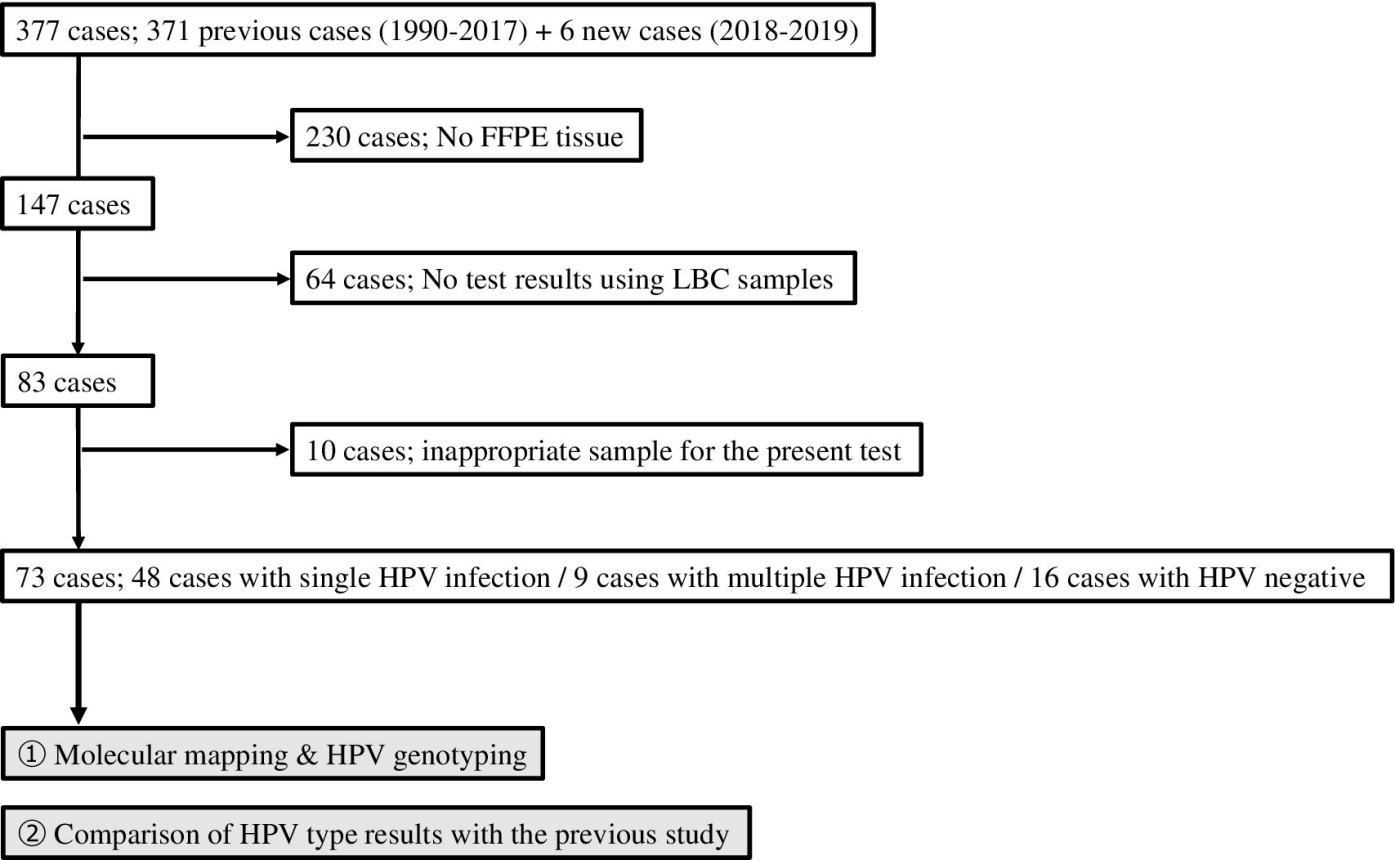
Study flow chart. ICC cases (*n* = 371) from 1990 to 2018 in the previous study [22] and six cases from 2018 to 2019 underwent HPV genotyping. Among those cases, 230 were excluded due to the unavailability of tissue samples, as these cases were from another hospital. Among 147 cases, 64 (1990–2004) were excluded, as there were no data on HPV type from LBC samples. Of 83 cases, 10 were excluded due to inappropriate samples. Finally, 73 cases were investigated in this study. A total of 48 ICC cases with single HPV infection, nine with multiple HPV infections, and 16 HPV-negative ICCs were examined. HPV genotyping was determined using a combination of a molecular-mapping procedure and uniplex *E6/E7* PCR.

This study was approved by the Clinical Ethics Committee of Kanazawa Medical University (approval no. I408). Written informed consent to participate was obtained from all patients. Fig 1 shows flow chart of the present study. Samples of 371 ICC cases were obtained from a multi-center study conducted between 1990 and 2017, and samples from another six ICC cases were obtained from the hospital of Kanazawa Medical University from 2018 to 2019. In the previous study, these 371 ICC cases were determined by HPV genotyping using fresh tissue or liquid-based cytology (LBC) samples by Genosearch-31 + 4 or Genosearch-31 [22]. Genosearch-31 (MBL, Nagoya, Japan), which is a commercially available test designed as a type-specific multiple-primer-based PCR targeting the *L1* DNA of 31 HPV types, including 13 high-risk types (Group 1+2A), six possible high-risk types (Group2B) (HPV26/53/66/70/73/82), and 12 low-risk types (HPV6/11/42/44/54/55/61/62/71/84/89/90), was used in the present study for HPV typing using LBC samples.

Genosearch-31+4 is a modified version of Genosearch-31 and capable of detecting four additional possible high-risk HPV types (HPV30/34/67/69). These assays are able to detect 1,000 copies of HPV DNA. Genosearch-31+4 was only used in HPV-negative cases in the first round of experiments. Among the 377 cases, 230 with no FFPE samples were excluded, because these had been come from other hospitals. Sixty-four cases from 1990 to 2004 with no HPV data from LBC samples excluded, as were 10 cases with inadequate FFPE tissue samples, given their concurrent treatment with chemoradiotherapy or chemotherapy were excluded. In the present study, we investigated 73 ICC specimens, including nine carcinoma cases infected with multiple HPV types, 16 HPV-negative cases, and 48 cases infected with a single HPV type. The histological diagnosis of ICC was made by two professional pathologists. There were 45 cases of squamous cell carcinoma (SSC), 11 of endocervical adenocarcinoma, usual type (usual ADC), seven unusual ADCs (4 mucinous carcinomas, 2 clear cell carcinomas, and one serous carcinoma), five ADSCC, four cases of neuroendocrine carcinoma (NEC; 2 large-cell NEC and 2 small-cell NEC), and one carcinosarcoma.

### Tissue microdissection from FFPE samples and DNA extraction

Cervical carcinoma tissue specimens were obtained between 2005 and 2019 at the time of surgery or punched biopsy during colposcopy. Five or more 10-μm-thick tissue slices were collected from FFPE tissue blocks. One section was stained with hematoxylin and eosin, and the remaining sections were stained with hematoxylin alone. The location of the carcinoma tissue was determined and identified through analysis of the tissue morphology and hematoxylin-staining patterns. p16 and Ki67 immunostaining was performed in some samples in order to define ICC areas. Carcinoma nest alone was then scratched out using a 24G needle, placed into a 1.5-mL microtube containing 50 μL of alkaline lysis reagent [25 mM NaOH, 0.2 mM EDTA (pH 12.0)], and incubated in a thermomixer (95°C, 15 min; 300, 300, 900, and 900 rpm). Equal amounts of neutralization reagent [0.04 mM Tris-HCL (pH 5.0)] were then added, and the samples were vortexed for 30 s. The samples were then centrifuged at 13,523*g* and stored in a freezer at −20°C until use. Approximately 1 μL to 5 μL of supernatant from the DNA-extraction solution was used for HPV genotyping.

### HPV genotyping using the *E6/E7*-based PCR method

HPV genotyping was performed using uniplex *E6/E7* PCR, which can detect 100 copies of DNA from each HPV type [24]. HPV DNA was amplified using specific primers for the *E6* or *E7* genes of the 13 high-risk types and 11 possible high-risk types, with *β-globin* used as an internal control gene. The PCR method was described in a previous study [24]. Briefly, PCR was performed in a T 100 Thermal Cycler (Bio-Rad Laboratories, Hercules, CA, USA) using the following program:10 min at 94°C, followed by 35 cycles of 30 s at 94°C, 30 s at 60°C, and 30 s at 72°C, with a final 5-min extension step. A total of 4 μL of each reaction solution was applied on a 2% agarose gel (Bio-Rad Laboratories) and electrophoresed in 1× TBE buffer. HPV-type-specific bands were visualized by SYBR Green I (Takara Bio, Shiga, Japan) staining and UV light. HPV genotype was determined using HPV-type-specific primers for the *E6* or *E7* gene of 24 HPV types, including 11 possible high-risk HPV types (HPV26/30/34/53/66/67/69/70/73/82/85) and 13 high-risk HPV types (HPV16/18/31/33/35/39/45/51/52/56/58/59/68). HPV16 *E6* gene primers were confirmed to have no mismatched sequences with HPV16 *E6* variants, except for some African variants (B4, C3, and C4; data not provided).

### Verification of amplified *E6* genes and the presence of HPV *E1*, *E2* and *L1* genes in cancer tissues

We verified HPV-typing results obtained via uniplex *E6/E7* PCR in some cancer cases showing discrepant results from previous typing and determined using *L1*-based PCR. Amplified *E6* DNA was purified with the MinElute PCR purification kit (QIAGEN. Hilden, Germany) and examined for the sequence using a 3500/3500xL system (Applied Biosystems, Foster City, CA, USA) in both directions using forward and the reverse primers.

Because *E6* DNA was undetected in many discordant cases, we applied the short-fragment *E6* PCR method to target HPV16 and HPV18 using the following primers:

s16E6-F-primer, TGACTTTGCTTTTCGGGATT

s16E6R-primer, CAACGGTTTGTTGTATTGCTG

s18E6-F-primer, GGTGCCAGAAACCGTTGAATCC

s18E6-R-primer, TACTTGTGTTTCTCTGCGTCGTTG

To assess the integration status of HPV genes in some ICC samples, we determined the presence of the *E1* and *E2* genes of HPV16, HPV18, and HPV67 using HPV-type-specific PCR. Fragmented *E1* and *E2* genes across the HPV16 and HPV18 genomes were amplified as previously reported [25]. The primer of the *E1* and *E2* genes for HPV67 were developed as follows.

HPV E1 and E2 primers for HPV67:

67E1a-F, AGGACCCGGAAGGTACAGAC

67E1a-R, CTCTACCTGCACCATTGTCTCTTG

67E1b-F, TCAAGAGACAATGGTGCAGGTAGA

67E1b-R, GTGTGTACAACGTTTGTGGCTTGA

67E1c-F, AATAACACCATCAGTAGCAG

67E1c-R, CAAAACTATGTTGCAGTACC

67E1d-F, GGATAGAAAGACTAACGGTACTG

67E1d-R, GTGGTCCGCATATTACCATAC

67E1e1-F, GGACCACCAAACACAGGGAA

67E1e1-R, CTAATTGGCACCACGTCCTTGA

67E1e2-F, GGACCACCAAACACAGGGAA

67E1e2-R, AAAGCACATTCAATGCGTCTCA

67E2a-F, GAGGACAAGGAAAACCATGGAG

67E2a-R, TCCCATATAGTCCACCTTTCCC

67E2b-F, GGGAAAGGTGGACTATATGGGA

67E2b-R, GCACTATAGGTGCAGTGTTAGG

67E2c-F, CCAGACTTCTGCAACAACTC

67E2c-R, TTAGCAGCACATATTGGCTT

Although we did this experiment, most of these genes were undetected in ICC cases showing discrepant results. *β-globin* was used as an internal control for DNA quality assessment but was also not detected in ~50% of cases, suggesting that DNA samples extracted from FFPE samples were likely fragmented. Therefore, we examined shorter fragments of *L1* and *E2 g*enes for HPV16, HPV18, and HPV67 using the following primers.

Short HPV-L1 fragment primers for HPV16, HPV18, and HPV67:

s16L1-F, GATACTACACGCAGTACAAATATGTC

s16L1-R, CCATGTCGTAGGTACTCCTTAAAG

s18L1-F, CGCAGTACCAATTTAACAATATGTGC

s18L1-R, CAAATCATATTCCTCAACATGTCTGC

s67L1-F, ACCCTAGCCAGCCTGGTACT

s67L1-R, AAAATCCATGCAGCCAAAAC

Short HPV-E2 (sE2) primers for HPV16, HPV18, and HPV67:

s16E2-F, ATGCGGGTGGTCAGGTAATA

s16E2-R, TCGCTGGATAGTCGTCTGTG

s18E2-F, GAAGGAAACCCTTTCGGAAC

s18E2 -R, GTATGCCATGTTCCCTTGCT

s67E2-F, GCACAAACAAAGGACGGAGT

s67E2-R, GGGGGTATGCGTACAGAGTT

PCR for the short fragments of the *L1*, *E2*, and *E6* genes for HPV16, HPV18, and HPV67 was performed similarly to uniplex *E6/E7* PCR using the following program: 10 min at 94°C, followed by 35 cycles of 30 s at 94°C, 30 s at 55°C to 60°C, and 30 s at 72°C, with a final 5-min extension step.

## Results

### Comparison of HPV types identified in cytology samples and archival tissue specimens analyzed by molecular mapping

Among the 377 ICC cases, 73 underwent reevaluation of HPV genotype by molecular mapping of FFPE tissue specimens (Fig 1). Among these 73 cases, 48 were previously identified as positive for a single HPV type, while 9 had multiple HPV types and 16 were HPV-negative. Liquid-based cytology (LBC) cervical cancer samples and a commercially available HPV test targeting the *L1* gene (Genosearch-31) were used in the previous study. In the present study, areas of carcinoma in FFPE tissue specimens from the same patients were excised using a manual microdissection procedure; HPV genotype was then determined using the *E6/E7*-PCR method.

Comparison of the HPV genotyping results showed that 52 cases (71.2%) had concordant results for HPV type, whereas the other 21 cases (28.8%) were not in agreement. Among the discordant cases, 6 (28.6%) exhibited partial concordance because one of the previously reported multiple HPV types was detected in the present analysis (Table 1). Additionally, complete discordance was observed in 15 cases (71.4%), among which 8 that were previously HPV-negative (53.3%) now harbored a single HPV type in the carcinoma lesion (Table 1). Furthermore, five cases, including three with multiple HPV types and two with a single HPV type, were now positive for other HPV types (Table 1). Two cases previously positive for low- and possible high-risk HPV types (HPV6/53) were now negative (not shown in Table 1).

**Table 1:** Cervical cancer cases that exhibited discordant HPV type results, and the presence of *E1*, *E2*, *L1*, and *E6* genes according to HPV type.

				*E1*					*E2*			*L1*	*E2*	*E6*		IC
				*E1a*	*E1b*	*E1c*	*E1d*	*E1e*	*E2a*	*E2b*	*E2c*	*sL1*	*sE2*	*E6*	*sE6*	*β-globin*
**Case No.**	**Discordant results (negative to positive)**	**LBC (GS31)**	**Microdissected FFPE (*E6/E7* PCR)**													
1	Adenosquamous carcinoma	Negative	HPV16	**-**	**-**	**-**	**-**	**+**	**-**	**-**	**+**	**+**	**+**	**-**	**+**	**+**
9	Squamous cell carcinoma	Negative	HPV16	**-**	**-**	**-**	**-**	**-**	**-**	**-**	**-**	**+**	**+**	**-**	**+**	**-**
10	Squamous cell carcinoma	Negative	HPV67	**+**	**+**	**+**	**-**	**-**	**-**	**-**	**-**	**+**	**+**	**+**	**NE**	**+**
14	Squamous cell carcinoma	Negative	HPV16	**-**	**-**	**-**	**-**	**+**	**-**	**-**	**-**	**+**	**+**	**-**	**+**	**-**
44	Large cell neuroendocrine carcinoma	Negative	HPV16	**-**	**-**	**-**	**-**	**-**	**-**	**-**	**+**	**+**	**+**	**-**	NE*	**-**
56	Squamous cell carcinoma	Negative	HPV16	**-**	**+**	**+**	**+**	**+**	**-**	**-**	**+**	**+**	**+**	**+**	**+**	**+**
61	Large cell neuroendocrine carcinoma	Negative	HPV16	**-**	**-**	**-**	**-**	**-**	**-**	**-**	**-**	**+**	**+**	**-**	**-**	**+**
77	Squamous cell carcinoma	Negative	HPV67	**-**	**-**	**-**	**-**	**-**	**-**	**-**	**-**	**+**	**+**	**-**	**NE**	**-**
**Case No.**	**Discordant results (different HPV type)**	**LBC (GS31)**	**Microdissected FFPE (*E6/E7* PCR)**													
22	Small cell neuroendocrine carcinoma	HPV54, 58	HPV16	**-**	**-**	**-**	**-**	**-**	**-**	**-**	**-**	**+**	**+**	**-**	**-**	**-**
31	Adenosquamous carcinoma	HPV52	HPV18	**-**	**-**	**-**	**-**	**-**	**-**	**-**	**-**	**+**	**-**	**-**	**+**	**-**
49	Squamous cell carcinoma	HPV58	HPV16	**-**	**-**	**-**	**-**	**-**	**-**	**-**	**+**	**+**	**+**	**-**	**+**	**-**
64	Squamous cell carcinoma	HPV6, 52, 55	HPV67	**+**	**+**	**+**	**+**	**+**	**+**	**+**	**+**	**+**	**+**	**+**	**NE**	**+**
73	Squamous cell carcinoma	HPV51, 52	HPV16	**-**	**+**	**-**	**-**	**-**	**-**	**-**	**-**	**+**	**+**	**-**	**+**	**+**
**Case No.**	**Concordant results (different HPV type)**	**LBC (GS31)**	**Microdissected FFPE (*E6/E7* PCR)**													
C1	Squamous cell carcinoma	HPV16	HPV16	**+**	**+**	**-**	**-**	**-**	**-**	**+**	**+**	**+**	**+**	**+**	**NE**	**+**
C2	Squamous cell carcinoma	HPV16	HPV16	**+**	**+**	**+**	**+**	**+**	**+**	**+**	**+**	**+**	**+**	**+**	**NE**	**+**
C3	Squamous cell carcinoma	HPV16	HPV16	**+**	**+**	**+**	**+**	**+**	**+**	**+**	**+**	**+**	**+**	**+**	**NE**	**+**
C4	Adenocarcinoma	HPV18	HPV18	**+**	**+**	**+**	**-**	**-**	**-**	**-**	**-**	**+**	**-**	**+**	**NE**	**+**
C5	Adenocarcinoma	HPV18	HPV18	**+**	**+**	**-**	**+**	**+**	**-**	**-**	**-**	**+**	**+**	**-**	**+**	**+**
**Case No.**	**Partially concordant results (multiple to single)**	**LBC (GS31)**	**Microdissected FFPE (*E6/E7* PCR)**													
21	Endocervical adenocarcinoma, usual type	HPV6, 16, 59	HPV16													
60	Squamous cell carcinoma	HPV67, 18	HPV18													
74	Squamous cell carcinoma	HPV16, 66, 82	HPV82													
75	Adenosquamous carcinoma	HPV52, 59	HPV59													
76	Endocervical adenocarcinoma, usual type	HPV18, 53	HPV18													
80	Squamous cell carcinoma	HPV56, 59	HPV56													

s*L*1, s*E*2, s*E*6, short-fragment PCR; IC, internal control; NE, not examined; NE*, not examined because samples were unavailable; +, positive gene; -, negative gene; HPV, human papillomavirus; LBC, liquid-based cytology; FFPE, formalin-fixed paraffin-embedded; PCR, polymerase chain reaction

Among the 15 cases that exhibited discordance, 9 (60.0%) were positive for HPV16 in this study, while 3 (20.0%) were positive for HPV67, 1 (6.7%) was positive for HPV18, and 2 had no HPV. HPV16 and HPV67 were the two most common HPV types missed by the previously employed HPV test targeting the HPV *L1* gene in cervical LBC samples. Notably, all HPV-positive cancer cases harbored a single HPV type; all were high-risk or possible high-risk HPV types.

### HPV genotype in cancerous and other areas of archival tissue specimens

**Fig 2:**
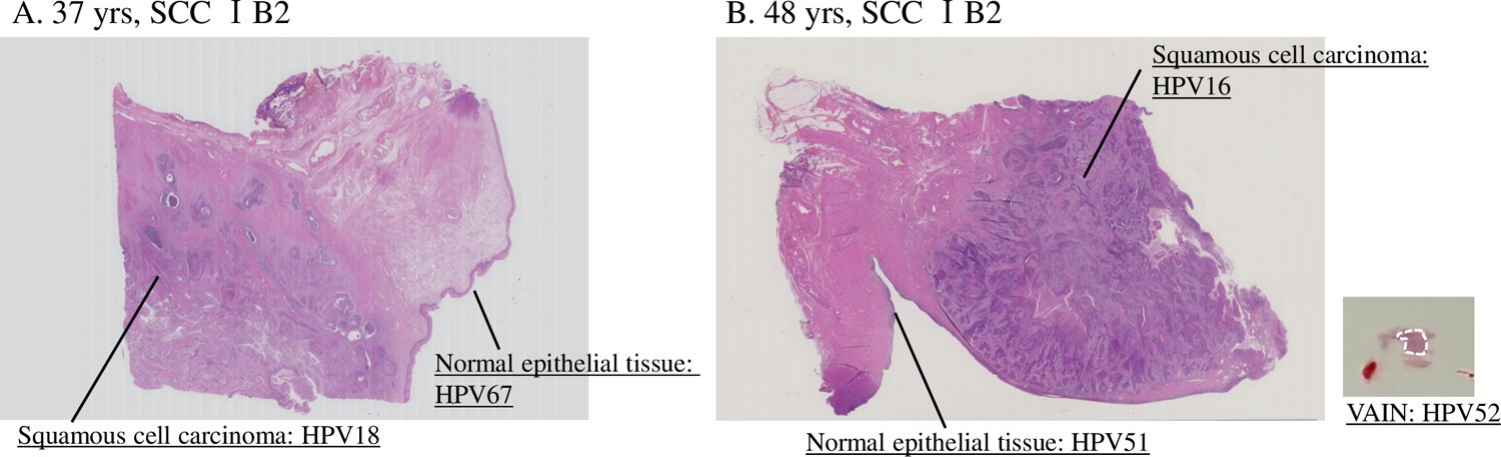
Histological findings and areas of microdissection in representative cases. (A) Case #60 (aged 37 years; SCC stage 1B2, positive for HPV18 and HPV67 in the previous study according to analysis of LBC samples and an *L1-*based HPV test). Upon reexamination by uniplex *E6/E7* PCR, HPV18 was detected in the SCC lesion, while HPV67 was detected in normal squamous epithelium. (B) Case #73 (aged 48 years; SCC stage 1B2, positive for HPV51 and HPV52 in the previous study). Reexamination revealed HPV16 in the SCC lesion, HPV51 in normal cervicovaginal epithelium, and HPV52 in VAIN1.

Among six cases that exhibited partial discordance (multiple to single) (Table 1), one case (#60) previously positive for HPV18 and HPV67 was now positive for HPV18 in a squamous cell carcinoma (SCC) sample, while HPV67 was detected in normal cervical epithelium near the cancer nest (Table 1 and Fig 2A). Examinations of five cases with completely discordant results (different HPV type) revealed that case #31, which was positive for HPV52 in the previous study, was positive for HPV18 in an ADSCC tissue sample. In this case, HPV18 was detected in both SCC and ADC components, while normal squamous cell epithelium was HPV52-positive. Additionally, case #73, in which HPV51 and HPV52 had been detected previously, was positive for HPV16 in a cancer tissue sample (Fig 2B). Moreover, HPV51 was identified in normal cervicovaginal epithelium, whereas HPV52 was detected in a low-grade vaginal lesion (VAIN1). This case had a unique clinical history: after two cervical conizations of high-grade intraepithelial lesions (CIN3), the patient had been continuously negative during > 10 years of annual check-ups using a Pap test. This clinical history suggests that ICC developed in deeper areas of the cervical canal after a long period of latency. We could not confirm the location of the HPV types previously detected in three cases (#22, #49, and #64) because insufficient tissue was available from patients who had undergone radiation therapy.

### Verification of *E6/E7* PCR assay validity and integration statuses of HPV genes in cases that exhibited disagreement in HPV type

**Fig 3:**
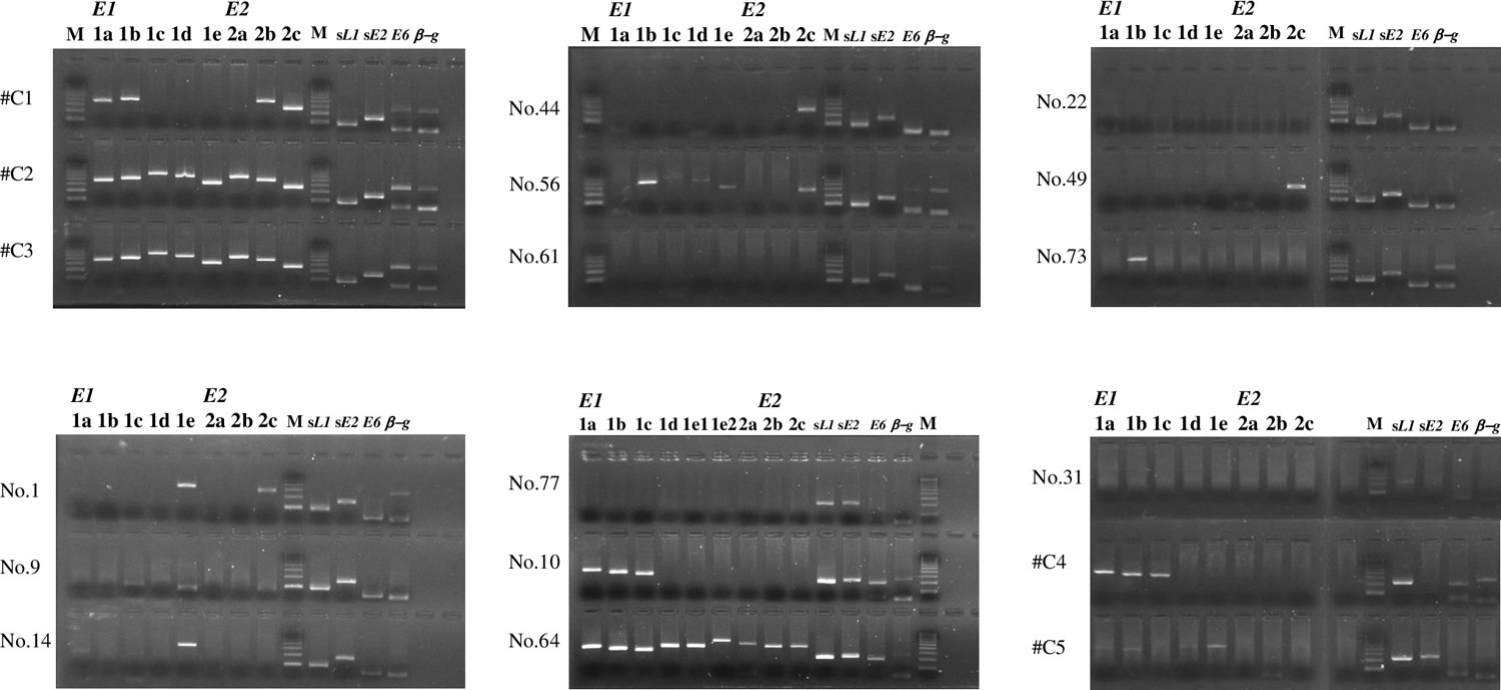
Detection of fragmented *E1* and *E2* genes and short fragments of the *L1*, *E2*, and *E6* genes. A 2% agarose gel was used to resolve five *E1* fragments (*E1a*, *E1b*, *E1c E1d*, and *E1e*) and three *E2* fragments (*E2a*, *E2b*, and *E2c*) covering the entire *E1* and *E2* genes of HPV16 and HPV18, as well as six *E1* fragments (*E1a*, *E1b*, *E1c*, *E1d*, *and E1e1+E1e2*) and three *E2* fragments (*E2a*, *E2b*, and *E2c*) covering the entire *E1* and *E2* genes of HPV67, short-fragment *L1* and *E2* genes of HPV16, HPV18, HPV67, *E6* gene (amplified via uniplex *E6/E7* PCR) of HPV16, HPV18, and HPV67, and *β-globin*. The *sE6* genes of HPV16, HPV18, and HPV67 are not shown in the figure. M: DNA marker; *β-g*: *β-globin*; #1, #9, #14, #22, #44, #49, #56, #61, #73: HPV16; #31: HPV18; #10, #64 and #77: HPV67; C1–C3: HPV16 controls; C4 and C5: HPV18 controls.

We identified a single HPV type in 19 cases that exhibited discordant and partially concordant results for HPV type (Table 1). To verify the HPV typing results in 13 discordant cases, we investigated the sequences of *E6* genes for HPV16, HPV18, and HPV67 in these samples; however, the *E6* genes for these HPV types generally were not amplified in the second round of experiments using uniplex *E6/E7* PCR (Fig 3). Additionally, *β-globin*, used as the internal control, was undetected in 7 of 13 discordant cases; this finding suggested that DNA samples extracted from FFPE specimens had been fragmented during the second round of testing. We then developed a PCR method to detect shorter fragments of the *E6* genes of HPV16 and HPV18, which identified a sequence for each target in seven of nine cases. Thus, two cases were negative, while no sample was available in one other case. Unexpectedly, all of these cases exhibited neuroendocrine carcinoma (NEC).

To determine the status of HPV gene integration into the host genome, we investigated the preservation statuses of the *E1* and *E2* genes in 13 discordant cases and in 5 control cases with concordant results (3 HPV16-positive and 2 HPV18-positive cases) (Table 1 and Fig 3). All or part of the *E1* and *E2* fragments were undetected in 12 of the 13 discordant cases, while such gene deletions were observed in one of three HPV16 controls and two HPV18 controls (Table 1 and Fig 3). The only case with preserved *E1* and *E2* genes was an HPV67-positive case. As suggested for *E6*, the fragmentation of *E1* and *E2* in FFPE specimens might have influenced this result. We then attempted to detect shorter *L1* and *E2* genes (< 200 bp) for HPV16, HPV18, and HPV67 using type-specific PCR. The *L1* genes of the HPV types were detected in all 13 cases, whereas *E2* was detected in 12 of the 13 cases; there was no band in 1 HPV18-positive case (#31).

### HPV genotype and histological classification

Table 2 shows the prevalence of HPV types according to histological classification. All SCCs were positive for HPV, compared to 65.2% (15/23) of the ADC and ADSCC cases. The most common HPV types belonged to the Alpha-9 group (HPV16/31/52/67; n = 48), followed by the Alpha-7 (HPV18/59/68; n = 13), Alpha-6 (HPV56; n = 1), and Alpha-5 (HPV82; n = 1) groups. One HPV67-positive case showed no response to chemotherapy and died of cervical cancer. The HPV82-positive case was diagnosed with basaloid SCC, and HPV67 and HPV82 were classified as possible high-risk types. Furthermore, 10 of 11 endocervical ADCs of the usual type were positive for HPV16 or HPV18, while 3 of 5 (60%) ADSCCs were positive for HPV16 or HPV18. The other two cases (40%) were positive for HPV59 or HPV68, which belong to the Alpha-7 group. The cancer tissue positive for HPV68 had a unique morphology resembling mucoepidermoid carcinoma. Three of four NECs were positive for HPV16; ADCs of other histological types (e.g., mucinous [gastric type], serous, and clear cell types) and carcinosarcoma were negative for HPV.

**Table 2:** Histology and HPV types.

		All ICC		SCC		ADSCC		All ADC		ECX, usual		ADC, others		NEC		Others	
	HPV group	No.	%	No.	%	No.	%	No.	%	No.	%	No.	%	No.	%	No.	%
HPV16	α-9	36	49.3%	28	60.9%	1	20.0%	4	22.2%	4	36.4%	0	0.0%	3	75.0%	0	0%
HPV18	α-7	11	15.1%	3	6.5%	2	40.0%	6	33.3%	6	54.5%	0	0.0%	0	0.0%	0	0%
HPV31	α-9	3	4.1%	3	6.5%	0	0.0%	0	0.0%	0	0.0%	0	0.0%	0	0.0%	0	0%
HPV52	α-9	6	8.2%	6	13.0%	0	0.0%	0	0.0%	0	0.0%	0	0.0%	0	0.0%	0	0%
HPV56	α-6	1	1.4%	1	2.2%	0	0.0%	0	0.0%	0	0.0%	0	0.0%	0	0.0%	0	0%
HPV59	α-7	1	1.4%	0	0.0%	1	20.0%	0	0.0%	0	0.0%	0	0.0%	0	0.0%	0	0%
HPV67*	α-9	3	4.1%	3	6.5%	0	0.0%	0	0.0%	0	0.0%	0	0.0%	0	0.0%	0	0%
HPV68	α-7	1	1.4%	0	0.0%	1	20.0%	0	0.0%	0	0.0%	0	0.0%	0	0.0%	0	0%
HPV82*	α-5	1	1.4%	1	2.2%	0	0.0%	0	0.0%	0	0.0%	0	0.0%	0	0.0%	0	0%
Negative		10	13.7%	0	0.0%	0	0.0%	8	44.4%	1	9.1%	7	100.0%	1	25.0%	1	100.0%
Total No.		73		45		5		18		11		7		4		1	
	; Alpha-7 group																
	; Alpha-5,6 and 9 groups																

HPV, human papillomavirus; ICC, invasive cervical cancer; SCC, squamous cell carcinoma; ADSCC, adenosquamous carcinoma; ADC, adenocarcinoma; NEC, neuroendocrine carcinoma; Other, other histology (carcinosarcoma)

## Discussion

The aim of this study was to investigate oncogenic HPV types in Japanese cervical cancer patients. Previous studies reported that 93–99.7% of ICC cases were positive for HPV [17, 26]. Furthermore, numerous studies have reported multiple HPV types in cervical premalignant lesions and cancers [27, 28]. In our previous study, 81.5% of ICC cases were positive for a single HPV type, 9.3% were positive for multiple HPV types, and 9.2% were negative for HPV [22]. It is critical that the oncogenic type is identified accurately in cases with multiple HPV types. Quint *et al*. [29, 30] detected only one HPV type in cervical and anal epithelial neoplasias using laser capture microdissection coupled with PCR genotyping. Snow *et al*. [31] reported that DNA extraction from FFPE tissue by manual microdissection, followed by molecular mapping, was an accurate and cost-effective approach. In the present study, we used a similar method to determine HPV genotypes. Preliminary experiments suggested that manual microdissection and uniplex *E6/E7* PCR were effective and accurate for determining HPV in cervical and vaginal tissue specimens [32], as well as single-cell samples [33].

In the present study, all 45 SCC cases, including 9 previously shown to harbor multiple HPV types (Table 1), were positive for one HPV type; the most common type was HPV16, followed by HPV52 and HPV18/HPV31/HPV67. Among 18 ADC cases, 10 were positive for one HPV type, the most common of which was HPV18 (followed by HPV16). Eight ADCs were HPV-negative, among which four were mucinous carcinomas, two were clear cell carcinomas, and one was serous carcinoma; there was one case of endocervical ADC (usual type). Previous studies reported that most (> 90%) SCCs are HPV-positive; moreover, 72–90% of endocervical ADCs are HPV-positive, whereas the other ADC types are HPV-negative [34–36]. The present study clearly demonstrated that almost all cervical SCCs and endocervical ADCs (usual type) were induced by high-risk or possible-high-risk HPV types, whereas other ADCs (e.g., serous, clear cell, and mucinous [gastric] types) were not. Furthermore, the SCC cases were positive for HPV16, HPV18, Alpha-9 types (HPV31/52/67), Alpha-5 types (HPV82), and an Alpha-6 type (HPV56). In contrast, ADC and ADSCC cases were positive for HPV16, HPV18, and Alpha-7 types (HPV59/68). These findings are consistent with our previous results [22]. A meta-analysis by Gary *et al*. [37] indicated that Alpha-7 HPV types have a greater tendency to induce ADC.

Henceforth, the primary HPV test will be incorporated into cervical cancer screening programs worldwide; it is more sensitive than the Pap test and shows similar specificity. However, there are some limitations to the HPV test because HPV-negative cancer exists in some geographical areas. Furthermore, possible high-risk HPV types such as HPV67 and HPV82, identified as a single type in some ICC tissues, cannot be detected using current commercial HPV tests [21]. The International Agency for Research on Cancer defines these HPV types as Group 2B carcinogens because they were rarely detected in isolation in large epidemiological studies of ICC [23]. Halec *et al*. [38] reported that the HPV26, HPV53, HPV66, HPV67, HPV69, HPV73, and HPV82 types should be considered high-risk because the expression patterns of certain biomarkers in these types were similar to the expression patterns of those biomarkers in invasive carcinomas previously linked to high-risk HPV types. Arbyn et al. reported HPV26, 53, 66, 67, 68, and 73, occurring in isolation, in 2.6% of cervical cancer cases. However, they concluded that there is no need to include these HPV types in HPV screening tests or vaccines because of their low prevalences [39]. In Japan, possible high-risk HPV types such as HPV53, HPV66, HPV67, HPV69, and HPV82 have been identified in ICC [22, 40]. Except for HPV66, these types are undetectable by any commercial HPV test, suggesting that some cases of cervical cancer and precursor lesions may be overlooked by current HPV tests, which will be used when primary HPV screening programs begin in Japan. In our previous study, 2.3% (7/306), and in this study, 2.9% (11/377) of cancer cases were positive for these possible high-risk HPV types [22], which might be lower than the percentage of cancer cases overlooked by the Pap test [41, 42]. However, a new HPV assay detecting common, possible-high-risk HPV types may perform better than currently available assays because prophylactic HPV vaccines is likely to reduce the prevalence of certain high-risk types (including HPV 16 and 18) in cervical cancer during the post-vaccination era.

Arryo *et al*. [8] reported that 48 of 92 cases classified as HPV-negative were subsequently identified as HPV-positive; HPV33 was the most frequent HPV type (29.2%) among HPV-negative carcinomas [8]. In the present study, 8 of 16 cervical cancers were previously false-negatives; the most common type among those cases was HPV16 (6/8), followed by HPV67 (2/6). HPV67 could not be detected by previous HPV tests (Genosearch-31) or a commercial HPV test [21].

In the present study, a single HPV type was identified in 13 of 21 cases with results that differed from findings in a previous study. For HPV genotyping, the previous study used LBC samples and an *L1*-based PCR test, whereas the present study used FFPE specimens and an *E6/E7*-based PCR test. To elucidate the reason for the discrepant results, we determined the status of HPV gene integration into the host genome according to the presence or absence of the *E1* and *E2* genes, considering that these genes are frequently deleted in some cervical cancers (a marker of HPV gene integration) [25]. The present study showed that all or parts of *E1* and *E2* were undetected in 12 of 13 discordant cases, as well as 3 of 5 controls. Moreover, these genes were preserved in two of three controls with HPV16, but not in either of the two controls with HPV18. Further analysis revealed the shorter *L1*, *E2*, and *E6* genes in all cases, except for *E2* in an HPV18 ADSCC case and *E6* in two NEC cases. HPV gene integration is unlikely to be associated with the discrepant results observed in the present study, considering that the *L1* gene (i.e., the target of previous HPV assays) was identified in all discordant cases. HPV gene integration might be responsible for the maintenance of low copy numbers of the HPV genome in cancer tissue [43]. Another possible reason is the difference in sensitivity between the two assays, where the detection limit of the previous assay is 1,000 copies of the HPV genome versus 100 copies in the current assay. Sample type (LBC vs. tissue specimens) might also influence outcomes; necrotic debris or abundant inflammatory cells in LBC samples from aggressive cancer cases would reduce PCR efficacy. For example, the two NEC cases in the present study (both advanced and highly aggressive tumors) were HPV-negative when targeting the *E6* gene during the second round of testing.

We verified the sequences of *E6* genes for each HPV type in the FFPE tissue specimens. Unexpectedly, the *E6* gene, which was positive in the first round test, could not be amplified in most cases with discrepant HPV results from the findings according to the L1-based HPV test. The reason for this phenomenon is unclear. However, shorter *E2* and *E6* genes (100–200 bp) were amplified—and the sequences were confirmed—in almost all of these samples; the detection limit of target genes in FFPE was shorter than we had expected. In a previous study, it was stated that, “while FFPE blocks stored for years have been used successfully for DNA analysis, it is important to note that the length of amplifiable gene fragments may decrease over time” [44]. Therefore, we suspect that detection failure for the *E6* gene may occur because of DNA fragmentation during the interval between the first- and second-round tests.

The present study had some limitations. First, we used a small number of samples, which included a high proportion of HPV-negative samples and a high proportion of samples with multiple types of HPV. A larger sample size more representative of the general population will be needed to confirm the present findings. Second, the inclusion of poor-quality DNA in some FFPE samples may have skewed the results; it also precluded the derivation of clear conclusions regarding the HPV gene integration status in some cases.

In some studies, HPV16/18- and HPV31/45-positive women screened for cervical cancer appeared to be at higher risk of high-grade cervical lesions [45, 46]. Therefore, HPV genotyping may be important for the management of patients with premalignant cervical diseases. Our findings clearly indicate that HPV typing tests using micro-dissected tissue are superior to tests that use LBC samples.

## Conclusion

The combination of cancer microdissection and application of *E6/E7*-based HPV PCR represents an improved approach for detection of oncogenic HPV types in cancer. The behaviors of specific HPV types have been clarified in this study: HPV16 and 18 are likely to induce both SCC and ADC, while HPV types belonging to the Alpha-5 (HPV82), -6 (HPV56), and -9 (HPV31/52/67) groups predominantly induce SCC. Finally, Alpha-7 HPV types (HPV59/68) induce ADC or ADSCC. Additionally, we found that all SCCs and usual-type ADCs were positive for high-risk HPV types, suggesting that HPV screening is a useful approach for the detection of these cancers and associated premalignant lesions. Moreover, some ICCs were induced by possible high-risk types, such as HPV67 and HPV82, suggesting that the choice of HPV test is crucial for accurate cervical cancer screening; current commercial HPV tests might be limited in this regard. Accurate HPV typing based on tissue samples may gain greater clinical importance in the future.
